# Complete Genome Sequence of Human Respiratory Syncytial Virus from Lanzhou, China

**DOI:** 10.1128/genomeA.00739-17

**Published:** 2017-08-24

**Authors:** Chuanfeng Zhu, Shengfang Fu, Xv Zhou, Li Yu

**Affiliations:** a Lanzhou Institute of Biological Products Co., Ltd., Lanzhou, China; b Shanghai Institute of Biological Products Co., Ltd., Shanghai, China

## Abstract

A complete genome of human respiratory syncytial virus was sequenced and analyzed. Phylogenetic analysis showed that the full-length human respiratory syncytial virus (HRSV) genome sequence belongs to gene type NA1. We sequenced the genome in order to create the full-length cDNA infectious clone and develop vaccines against HRSV.

## GENOME ANNOUNCEMENT

Human respiratory syncytial virus (HRSV) is the most important viral agent of serious respiratory tract illness in infants and children worldwide (1, 2). Nearly all children are infected by HRSV at least once by the age of 2 years (3). HRSV is an enveloped, nonsegmented, single-stranded, negative-sense RNA virus of the genus *Pneumovirus*, family *Paramyxoviridae*. Its genome has about 15,000 bp and 11 genes encoding for nonstructural protein 1 (NS1) and NS2, nucleocapsid protein (N), phosphoprotein (P), matrix protein (M), small hydrophobic protein (SH), attachment glycoprotein (G), fusion glycoprotein (F), transcription regulatory proteins M2-1 and M2-2, and a large polymerase (L) (1). Based on antigenic and genetic variability in the G gene, HRSV is categorized into two groups, A and B. Groups HRSV-A and HRSV-B were further subdivided into 11 (ON1, GA1 to GA7, SAA1, NA1, and NA2) and 20 (GB1 to GB4, BA1 to BA10, SAB1 to SAB4, URU1, and URU2) genotypes (4, 5), respectively. The HRSV complete genomic sequence information will benefit the understanding of the molecular evolution of HRSV and the development of vaccines.

In this study, swabs from outpatient children with respiratory disease symptoms in Lanzhou University Second Hospital were collected and transported immediately to our laboratory. RNA was extracted using an RNeasy minikit (Qiagen) and reverse transcribed into cDNA by M-MLV (Invitrogen). PCR was conducted to detect HRSV-positive samples. The positive samples were chosen to obtain the complete genome sequence. Briefly, 16 genomic segments were amplified to cover about 15 kb of the full-length genome using reverse transcription-PCR. The purified PCR products were ligated with pGEM-T Easy vector (Promega). The recombinant plasmids were identified by a restriction enzyme digestion method and then sequenced by a commercial sequencing service company. The obtained sequences were analyzed by sequence analysis software, and alignment was conducted using DNAman version 8.0 and MEGA version 4.0.

HRSV-A strain LZ01/09 was 15,204 nucleotides in length, including a 3′ leader and a 5′ trailer. The 11 open reading frames (ORFs) encoding viral proteins were deduced for the NS1, NS2, N, M, P, G, F, SH, M2-1, M2-2, and L genes. The contents of A, U, G, and C were 38.8%, 27.8%, 15.8%, and 17.7%, respectively. The nucleotide sequence identity of HRSV-A ranged from 94.9% to 96.7% compared with whole-genome information for some reference strains of HRSV-A. The deduced amino acid sequence identity ranged from 95.3% to 99.5%, in accordance with the corresponding ORFs of the reference strains, except those of G and M2-2. The phylogenetic analysis showed that HRSV-A strain LZ01/09 belongs to gene type NA1 of the HRSV-A group and is clustered together with GER 0825/05/06, GER 3897/06-07, GER 0897/06-07, Beijing A/04/07, Beijing A/04/58, and Chongqing A/08/03 (6). The HRSV genotype (HRSV-A NA1) was similar to those circulating during the same period in Cambodia, Japan, and Canada (4, 5, 7), suggesting that the genotype strain might be circulating globally. This study highlights the importance of global molecular epidemiological surveys of new strains of HRSV and provides a public health warning for possible outbreaks in the future.

### Accession number(s).

The whole-genome sequence of HRSV-A strain LZ01/09 has been deposited in GenBank under the accession no. KY782635.
